# The differences in sex ratio between sporadic and familial amyotrophic lateral sclerosis: a systematic review

**DOI:** 10.1007/s00415-026-13627-1

**Published:** 2026-01-21

**Authors:** Aafke Boomsma, Caitlin Doyle, Na Sai, Mary-Louise Rogers, Sang Hong Lee, Beben Benyamin

**Affiliations:** 1 Australian Centre for Precision Health, School of Public Health, Adelaide University, Adelaide, South Australia Australia; 2 South Australian Health and Medical Research Institute (SAHMRI), University of South Australia, Adelaide, Australia; 3 College of Medicine and Public Health, Flinders University, Adelaide, Australia; 4 Perron Institute for Neurological and Translational Science, Nedlands, Australia

**Keywords:** ALS, MND, Sex ratios, Diagnostic criteria, Meta-analysis, Systematic review

## Abstract

Amyotrophic lateral sclerosis (ALS) is more prevalent in males than in females. However, it is unclear whether the difference in sex ratio is observed similarly in sporadic compared to familial ALS. Here, we conducted a systematic review to investigate the differences in sex ratio between familial and sporadic ALS. Following the meta-analysis of observational studies in epidemiology (MOOSE) guidelines, this study searched Ovid MEDLINE, Embase, Emcare, SCOPUS and Cochrane databases. We used a random-effects meta-analysis to estimate sex ratios in a total of 9269 ALS patients (4135 female, 5134 male) across 20 included studies. We confirmed that ALS is more prevalent in males than in females (sex ratio: 1.25 (95%CI 1.14–1.37). However, when we stratified the analyses, the sex ratio was only different in sporadic ALS. Male-to-female ratios were 1.29 (95% CI 1.16–1.42) for sporadic ALS and 1.05 (95% CI 0.93–1.18) for familial ALS. A further analysis showed a pooled risk ratio of 1.07 (95% CI 1.00–1.15), indicating a 7% higher likelihood of males being diagnosed with sporadic rather than familial ALS. These findings highlight the importance of considering sex-specific factors in ALS research and clinical practice.

**Supplementary Information:**

The online version contains supplementary material available at 10.1007/s00415-026-13627-1.

## Introduction

Amyotrophic lateral sclerosis (ALS), also known Motor Neuron Disease (MND), refers to a group of rare neurodegenerative disorders [1, 2]. ALS primarily affects upper motor neurons in the brain and lower motor neurons in the brainstem and spinal cord [3], leading to progressive muscle weakness and paralysis, and typically results in a life expectancy of 3–5 years after diagnosis [4]. ALS is estimated to have an increased [5] global prevalence and incidence of 4.42 (95% CI 3.92–4.96) per 100,000 persons and 1.59 (95% CI 1.39–1.81) per 100,000 person-years, respectively [6]. A combination of genetic and environmental factors is thought to predispose risk to ALS, though its aetiology is unknown [7]. In about 10% of ALS cases, there is a clear pattern of familial inheritance, where more than half can be attributed to the mutation(s) in a single gene (familial ALS), such as *SOD1*, *C9orf72*, *TARDBP* and *FUS* [8]). The remainder of ALS cases show no clear pattern of familial inheritance. Still, a 40–50% heritability estimate suggests that multiple genetic variants with minor effects, combined with environmental factors, contribute to the disease liability in these apparent sporadic cases (e.g. Trabjerg et al. 2020 [9] and Ryan et al. 2019 [10]).

Sex ratio is expressed here as the ratio of males to females. Previous studies [4, 11–13] demonstrate that ALS is more prevalent in males than in females, although the exact sex ratio varies by geographic region and population. Most estimates place the ratio of males to females between ~ 1.3 [4] and 1.5 [3]. A study in the UK [11] reported that the sex ratio in ALS was significantly higher in younger (pre-menopausal) individuals (3.7) than in older age groups (1.2–1.4). Another study [14] found a progressive reduction of the sex ratio with increasing age. Interestingly, one study indicated a 1:1 male-to-female ratio in familial ALS, as well as a male preponderance of 2:1 and 1.5:1, respectively, in sporadic ALS [15, 16]. Sex-related differences in ALS have been studied [17] without any systematic reviews conducted on the differences between sporadic and familial ALS.

Frontotemporal Dementia (FTD) and ALS share some clinical, neuropathological and genetic features [18]. The shared genetic feature is the most frequent mutation in the *C9orf72* gene [19–22]. A previous meta-analysis focusing on sex differences in FTD and ALS [23] found a higher prevalence of female patients with a *C9orf72*-mutation in ALS, yet no difference in FTD. However, they found higher prevalence of female patients with FTD for the GRN mutation.

Understanding differences in sex ratios between familial and sporadic ALS may help identify sex-specific risk factors and these insights have important implications for both research and clinical practice. A clearer understanding of these differences may inform the design of clinical trials and assist clinicians in recognising potential sex-related patterns in the diagnosis of familial ALS and sporadic ALS. Therefore, this study aims to systematically review eligible studies to investigate the difference in sex ratios between familial and sporadic ALS cases.

## Methods

### Protocol registration

We followed the Preferred Reporting Items for Systematic Reviews and Meta-Analyses (PRISMA) 2020 flow diagram [24] in combination with the Meta-Analysis and Systematic Reviews of Observational Studies (MOOSE) 2021 [25], available in Supplementary Materials Table S1. We registered the protocol to PROSPERO International Prospective Register of Systematic Reviews (CRD42023384518) on 02 January 2023, with the last amendment made on 13 August 2024.

### Search strategy

We undertook a comprehensive search in Ovid MEDLINE, Embase, Emcare, SCOPUS and Cochrane databases as well as grey literature, and we performed a hand search as a follow-up from relevant identified studies. Efforts to include all available studies included intra-library requests and requesting full texts directly from authors through ResearchGate. One researcher (AB) designed the search strategy in collaboration with an experienced librarian and with input from two other reviewers (CD and BB). The full strategy, listing all search terms used, is available in Supplementary Materials Table S2.

### Review process

**Fig. 1:**
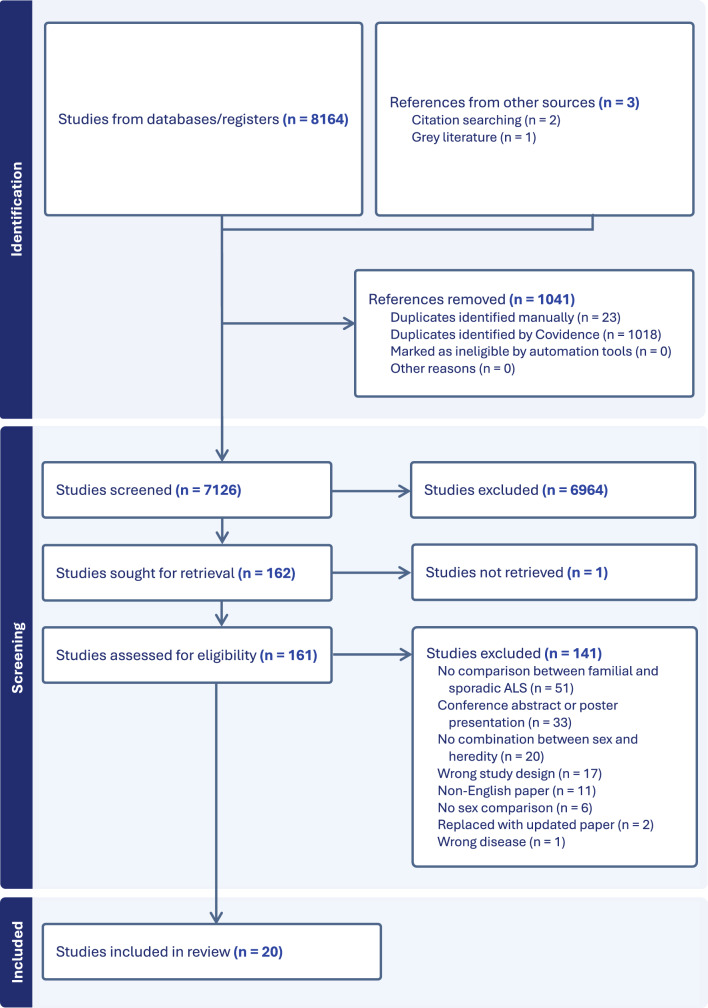
PRISMA flow diagram demonstrating study screening and inclusion

First, two reviewers (AB and CD or NS) independently screened search results for eligibility based on title, abstract, and keywords to remove clearly irrelevant studies (Fig. 1). Any conflicts between reviewers that occurred were resolved through consensus. Second, both reviewers (AB and CD or NS) independently screened full texts and discussed arising conflicts between reviewers, where a third reviewer (BB) resolved any remaining conflicts. Third, one reviewer (AB) performed data extraction and quality appraisal, with quality control performed by a second reviewer (BB). We performed the quality appraisal using the STROBE appraisal tool [26]. The software we used for screening, full text review, extraction and quality appraisal was Covidence [27]. Covidence is a web-based collaboration software platform that streamlines the production of systematic and other literature reviews.

Extracted data included: (1) Study design, (2) Birth Cohort (year), Mean age (years) ± SD of participants, (3) Ethnicities/ancestries, (4) Total number of participants, (5) numbers of familial and sporadic cases divided by females and males. Familial was defined differently in different studies, although definitions that involved a familial relationship were most common (13 out of 20, 65%).

### Inclusion/exclusion criteria

Table 1 gives the inclusion and exclusion criteria for this study. We included studies from database inception until 1 October 2025, peer-reviewed articles in the English language only and excluded conference abstracts, posters and unpublished studies. Included studies were required to: (1) have a human population of adults (aged ≥ 18 years) with either ALS or MND, (2) be classified as peer-reviewed studies, observational studies, population-based studies, epidemiological studies, longitudinal studies, cohort studies or cross-sectional studies, (3) include numbers of both sex (females and males) and ALS type (familial and sporadic) as well as numbers of sex per ALS type.

**Table 1:** Inclusion and exclusion criteria

Included	Excluded
Human studies	Animal studies
ALS and MND	MND’s that are not ALS
Type of publication: - Peer-reviewed studies, AND - Observational studies, OR - Population-based studies, OR - Epidemiological studies, OR - Longitudinal studies, OR - Cohort studies, OR - Cross-sectional studies	Type of publication: - Systematic Review - Meta-analysis - Non-English - Book chapters - Conference papers - Case reports - Clinical trials

### Definition of familial ALS

Although diagnostic criteria for familial ALS are outlined in the El Escorial criteria [28, 29], a consensus has not yet been reached on a standard definition of familial ALS among clinicians [30]. A definition for ‘probable familial ALS’ was proposed as “kindreds with one affected first- or second-degree relative” [31]. This definition was used in combination with a second, broader definition, including familial ALS as one first-degree relative with FTD, when the index case carried a GGGGCC hexanucleotide expansion in the first intron of *C9orf72* gene [32]. A complication for the analysis of ALS pedigrees is the identification of the *C9orf72* gene hexanucleotide expansions as a major cause of both ALS and frontotemporal dementia (FTD) [20, 33, 34]. A further complication is the increased identification of genes linked to ALS susceptibility [35]. In this review, we used the definition of familial ALS based on the definition used in the selected study. Out of 20 studies, 13 used a familial connection. Out of the 7 remaining studies, 3 used genetic mutations and 4 followed hospital records/diagnosis.

### Statistical analysis

We undertook exploratory analyses to investigate any differences in sex ratios between familial and sporadic cases of ALS. We also performed a meta-analysis using the analytical package Metafor in R [36]. We used random analyses due to heterogeneity in the diagnostic criteria of familial versus sporadic cases across the studies. Publication bias was investigated by testing for funnel plot asymmetry (t = 0.36, *p* = 0.73) and by visual inspection of a funnel plot Figure S2.

## Results

### Study characteristics

The initial search identified 8,167 studies. After removing duplicates and screening titles and abstracts, 161 full-text articles were assessed for eligibility. Of these, 20 studies met the inclusion criteria and were included in the review. The primary reason for exclusion at the full-text stage was due to non-reporting on the numbers of familial and sporadic cases separately (*n* = 51, 36.2%) (Fig. 1). Characteristics of the 20 studies included for review are in Table 2.

**Table 2:** Characteristics of included studies

Study ID	Title	Classification “familial”	Study design	Mean age (years) ± SD	Total number of participants
Baumgartner 2024 [47]	Genetic Landscape of Amyotrophic Lateral Sclerosis in Czech Patients	Family history	Cohort study	51.2 (19; 82)	15
Bozovic 2025 [48]	Transcranial Brain Parenchyma Sonographic Findings in Familial and Sporadic Amyotrophic Lateral Sclerosis	Gene mutation and/or family history	Cross sectional study	fALS 57.6 ± 11.8 sALS 60.0 ± 10.5	309
Corcia 2018 [49]	Phenotypic and genotypic studies of ALS cases in ALS-SMA families	Family history	Cohort study	58.7 (51–70) ± 5.7	15
Corcia 2025 [50]	Prevalence of SOD1 and C9orf72 Variants Among French ALS Population: The GENIALS Study	Family history	Cohort study	67 (59.0–73.0)	999
deAlcantara 2023 [51]	A comparative study of cognitive and behavioral profiles between sporadic and type 8 amyotrophic lateral sclerosis	Gene mutation (p.P56S), known as ALS8	Other: Comparative study	ALS8: 49 (43–58), sALS: 55 (52–61)	79
Drigo 2013 [52]	The incidence of amyotrophic lateral sclerosis in Friuli Venezia Giulia, Italy, from 2002 to 2009: a retrospective population-based study	Genetic test and family history	Cohort study	69 ± 6	262
Henden 2024 [53]	Short tandem repeat expansions in LRP12 are absent in cohorts of familial and sporadic amyotrophic lateral sclerosis patients of European ancestry	By diagnosis?	Cohort study	Unknown	643
Kotan 2020 [54]	Phenotypic and genotypic features of patients diagnosed with ALS in the city of Sakarya, Turkey	Genetic analyses on patients and family members	Cohort study	60.75 ± 10.25	55
Leighton 2019 [55]	Changing epidemiology of motor neurone disease in Scotland	Family history	Cohort study	Onset: 65.3 ± 11.6, Diagnosis: 66.8 ± 11.2	339
Li 1988 [56]	Comparison of sporadic and familial disease amongst 580 cases of motor neuron disease	Family history	Cohort study	sALS: 56 (13–87) ± 12.4/fALS: 52 (19–74) ± 14.3	580
Mitsi 2024 [57]	Genetic epidemiology of amyotrophic lateral sclerosis in Cyprus: a population-based study	Family history	Cohort study	58.51 ± 11 fALS: 55 ± 12.24 sALS: 59 ± 10.39	89
Mrkela 2025 [58]	The genetics of motor neuron disease in New Zealand	By diagnosis and family history	Cohort study	58.5 ± 12.3 fALS: 50.7 ± 14.3 sALS: 59.6 ± 11	149
Nalini 2008 [59]	Madras motor neuron disease (MMND): clinical description and survival pattern of 116 patients from Southern India seen over 36 years (1971–2007)	By diagnosis	Cohort study	15.8 ± 7.9	116
Norris 1993 [60]	Onset, natural history and outcome in idiopathic adult motor neuron disease	Family history	Cohort study	sALS: 57.8 fALS: 52.1 (25–74)	665
Palese 2019 [61]	Epidemiology of amyotrophic lateral sclerosis in Friuli-Venezia Giulia, North-Eastern Italy, 2002–2014: a retrospective population-based study	As state by (hospital) records	Other: retrospective population-based study	67.6 (20–90) ± 11.4	444
Ryan 2019 [10]	Lifetime Risk and Heritability of Amyotrophic Lateral Sclerosis	Parent–Offspring heritability (C9orf72-Negative)	Other: A prospective population-based parent–offspring heritability study	Onset: 64.9 ± 11.3 Diagnosis: 66.3 ± 11.3	1117
Shen 2024 [62]	Clinical and genetic characteristics of 1672 cases of amyotrophic lateral sclerosis in China: a single-center retrospective study	Family history	Cohort study	sALS 52.7 ± 11.2 fALS 49.3 ± 11.7	1672
Smith 1988 [63]	Motor neuron disease in the Rocky Mountain region	Family history	Other: Comparative study	sALS: 56.2 (17–85) ± 14/fALS: 50.6 (35–67) ± 14.4	116
Werneck 2007 [64]	A clinical epidemiological study of 251 cases of amyotrophic lateral sclerosis in the South of Brazil	Genetic testing	Cohort study	54.4 ± 12.3	251
Yamakawa 2022 [65]	Demographics, clinical characteristics, and prognostic factors of amyotrophic lateral sclerosis in Midwest	Family history	Cohort study	64.9 ± 11.5	1447

### Risk of bias

**Fig. 2:**
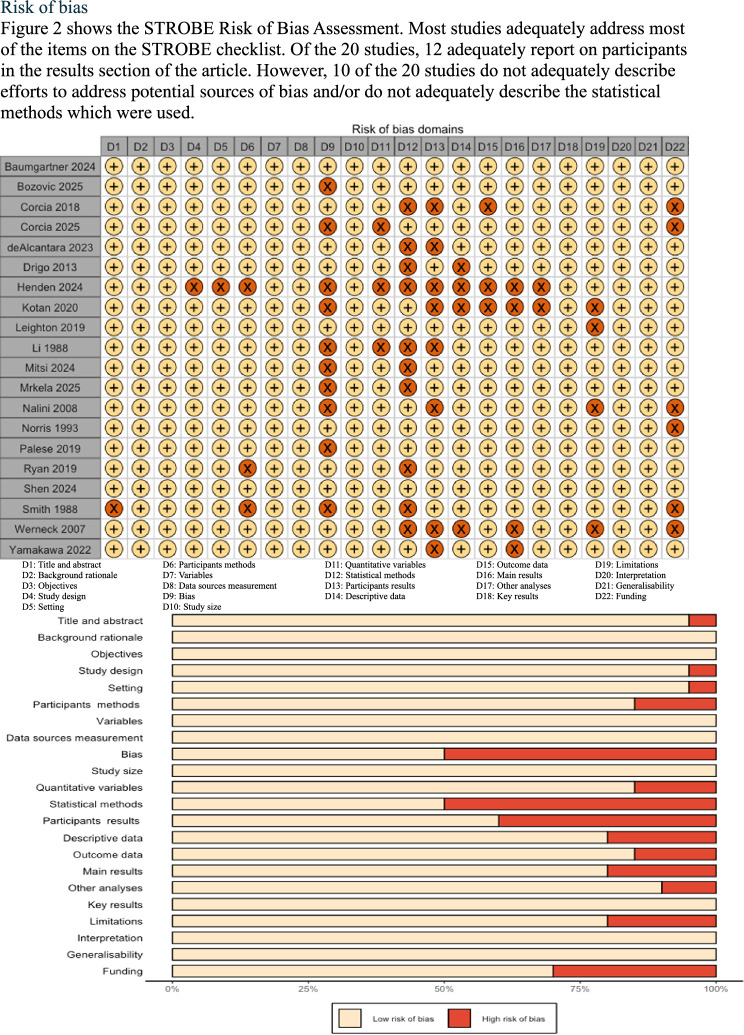
STROBE risk of bias assessment to appraise the quality of included studies

Figure 2 shows the STROBE Risk of Bias Assessment. Most studies adequately address most of the items on the STROBE checklist. Of the 20 studies, 12 adequately report on participants in the results section of the article. However, 10 of the 20 studies do not adequately describe efforts to address potential sources of bias and/or do not adequately describe the statistical methods which were used.

### Key findings

#### Study design

Of the 20 studies used in the meta-analysis 15 were cohort studies and 2 were comparative studies. There was also 1 cross-sectional study, 1 retrospective population-based study and 1 prospective population-based parent–offspring heritability study.

#### Ethnicities/ancestries

The ethnicity of participants was reported in 9 of 20 studies; 4 studies included participants of European ancestry (including Irish, Czech, and Greek-Cypriot), 2 from multiple ethnic backgrounds, 1 from individuals of South Indian ancestry, 1 from individuals of Chinese ancestry, and 1 primarily from individuals of Scottish ancestry.

#### Male-to-female ratio

Table 3 shows the number of individuals stratified by sex and pattern of inheritance (i.e. familial and sporadic), along with the corresponding sex ratios derived from the included studies. The sex ratio for familial cases ranges from 0.62 to 1.69 with a median of 1.00, while the sex ratio for sporadic cases ranges from 0.95 to 2.00 with a median of 1.31 (Supplementary Materials Figure S1). Our analyses show a different male: female ratio in familial cases of roughly 1 compared to sporadic cases of 1.29, suggesting sporadic ALS is more prevalent amongst males, while familial ALS affects the sexes equally.

**Table 3:** The number of individuals per sex for familial and sporadic heritability

Study ID	Familial			Sporadic		
	Male	Female	M:F Ratio	Male	Female	M:F Ratio
Baumgartner 2024	4	4	1:1	4	3	1.33:1
Bozovic 2025	14	17	0.82:1	152	126	1.21:1
Corcia 2018	6	6	1:1	2	1	2:1
Corcia 2025	45	40	1.13:1	495	422	1.17:1
deAlcantara 2023	17	12	1.42:1	12	8	1.5:1
Drigo 2013	6	5	1.2:1	126	125	1.01:1
Henden 2024	22	13	1.69:1	384	224	1.71:1
Kotan 2020	5	5	1:1	27	18	1.5:1
Leighton 2019	14	22	0.64:1	194	109	1.78:1
Li 1988	12	15	0.8:1	341	212	1.61:1
Mitsi 2024	8	13	0.62:1	36	32	1.13:1
Mrkela 2025	13	8	1.63:1	72	56	1.29:1
Nalini 2008	13	14	0.93:1	46	43	1.07:1
Norris 1993	29	23	1.26:1	364	249	1.46:1
Palese 2019	10	6	1.67:1	212	216	0.98:1
Ryan 2019	15	17	0.88:1	611	474	1.29:1
Shen 2024	48	41	1.17:1	785	798	0.98:1
Smith 1988	2	3	0.67:1	54	57	0.95:1
Werneck 2007	4	3	1.33:1	153	91	1.68:1
Yamakawa 2022	66	66	1:1	714	538	1.33:1

### Meta-analyses

#### Meta-analysis on all cases

**Fig. 3:**
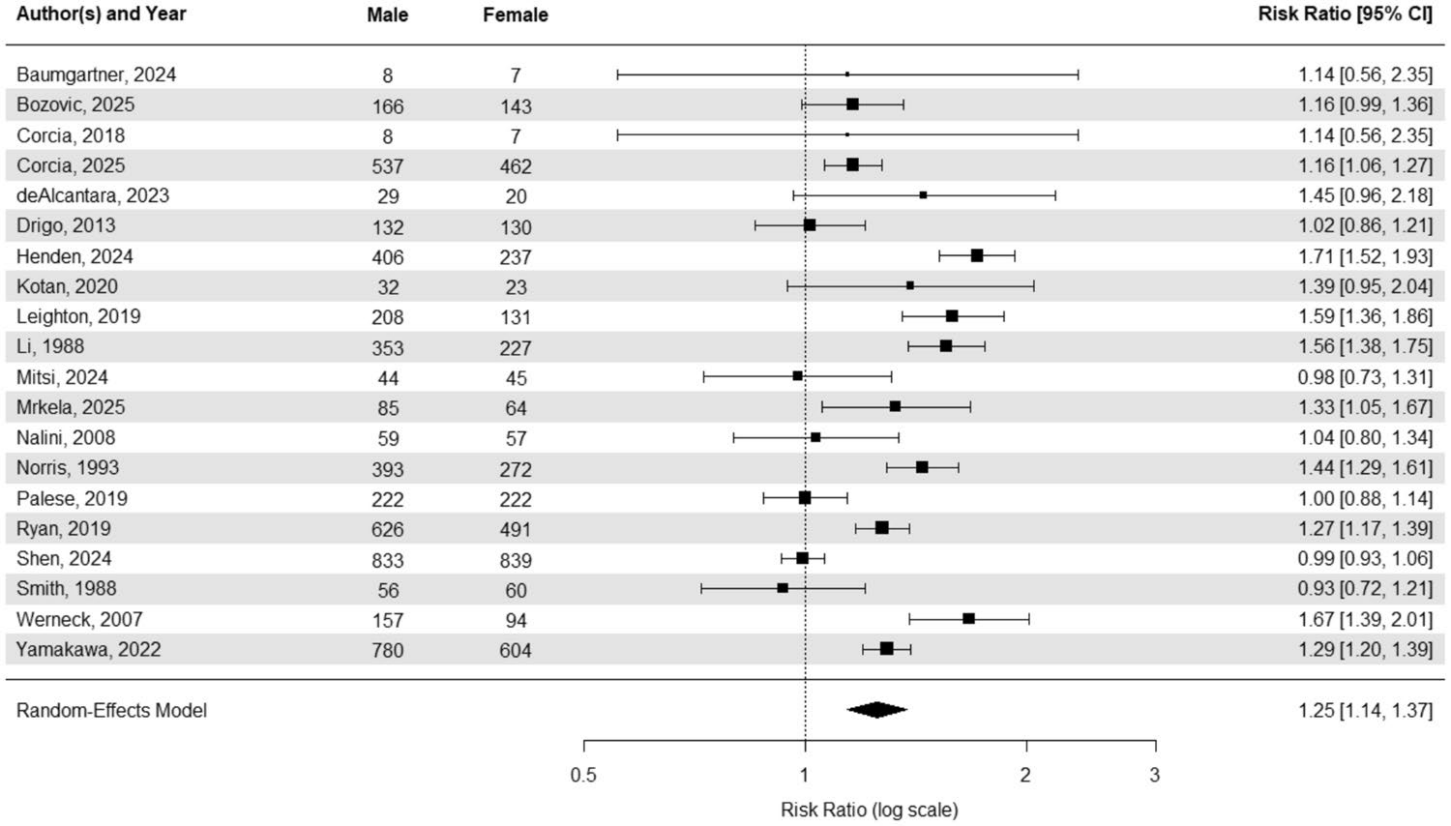
Forest plot of Sex Ratio and confidence interval for both sporadic and familial ALS cases

**Fig. 4:**
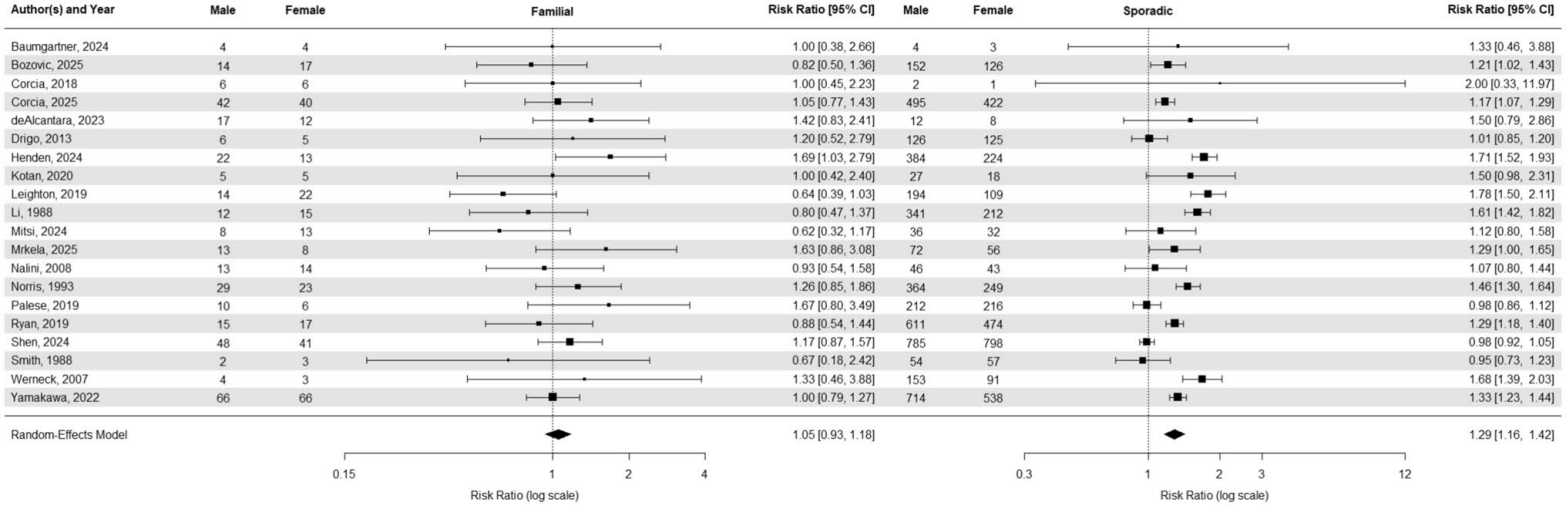
Forest plot of Sex Ratio and confidence interval for Familial ALS cases (left) and Sporadic ALS cases (right)

A random-effects model meta-analysis of combined sporadic and familial cases (Fig. 3) yields a male-to-female sex ratio of 1.25 (95% CI, 1.14–1.37). When we stratified the analyses into familial and sporadic (Fig. 4) cases only, the estimated sex ratios are 1.05 (95% CI 0.93–1.18) and 1.29 (95% CI 1.16–1.42), respectively.

#### Meta-analysis comparing the relative risk of males being diagnosed with Sporadic versus Familial ALS compared to females

**Fig. 5:**
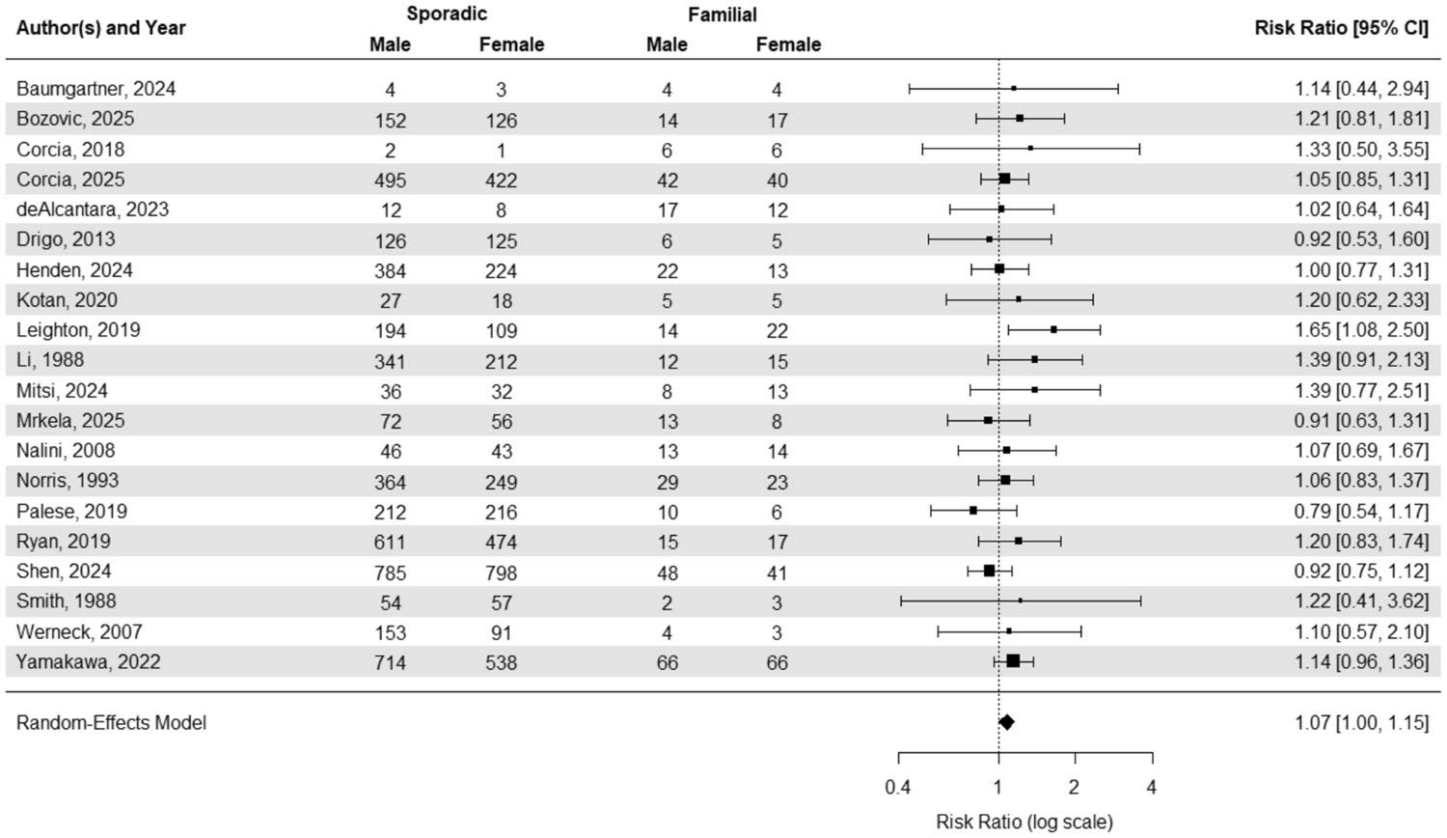
Forest plot of Sex Ratio and confidence interval comparing sporadic and familial ALS cases

We conducted a random-effects meta-analysis to estimate whether males are more likely than females to be diagnosed with sporadic rather than familial ALS (Fig. 5). The analysis yielded a pooled risk ratio of 1.07 (95% CI: 1.00–1.15), indicating that males have a 7% higher likelihood of being diagnosed with sporadic ALS relative to familial ALS when compared to females.

## Discussion

This systematic review investigated the difference in sex ratio between familial and sporadic ALS cases. To our knowledge, this is the first systematic review to estimate the sex ratio differences in combination with the aetiology of ALS (i.e. familial and sporadic). Our result (male-to-female ratio of 1.25) provides strong evidence to support the findings that ALS is more prevalent amongst males than females [4, 11–13]. However, our research further reveals that this difference is driven primarily by the sex ratio in sporadic cases (1.29), whereas familial ALS shows an approximately equal sex distribution (1.05).

The sex ratio of sporadic ALS is consistent with previous estimates between ~ 1.3 [4] and 1.5 [3]. Our results for both sporadic and familial ratios are consistent with the estimates mentioned in a previous study (1:1 in familial ALS and 1.5:1 in sporadic ALS) [16] as well as a study from Norway that found the sex ratio equalising in familial cases, indicating that genetic variants associated with ALS are not affected by gender [37]. Although our findings align with previous research, the different sex ratios between familial and sporadic ALS have not been systematically studied.

The higher male-to-female ratio that is only observed in sporadic ALS may indicate there are genetic or environmental factors that are specific to males, which make them uniquely predisposed to developing sporadic ALS. The prevalence of familial ALS is the same between males and females. Conversely, in sporadic ALS, non-genetic hazards and exposures that are considered male-specific (e.g. high levels of physical activity [38], smoking [39] and alcohol consumption [40]) may contribute to higher prevalence in males [41]. Another explanation could be misdiagnosis [42] or delayed diagnosis [43] of ALS in females. Family history and genetic testing might be factors contributing to a quicker and more accurate diagnosis of familial ALS. A population-based, case–control study in the Netherlands [44] amongst 881 participants found that each year longer of reproductive time span [HR 0.90 (*p* = 0.01)] and lifetime endogenous estrogen exposure [HR 0.96 (*p* = 0.025)] were associated with a longer survival of ALS patients. A more recent study [45] using 158 postmenopausal women had similar findings.

The primary limitation of this systematic review is that only 20 studies provided the information required to be included in this analysis. The primary reason for exclusion was the lack of stratification between familial and sporadic ALS cases. This could be a result of the lack of consensus among clinicians on the definition of familial ALS or the study focusing on topics other than the difference between familial ALS and sporadic ALS. A second reason for excluding studies was that they reported the numbers of participants for females and males as well as for sporadic and familial cases but did not provide a combination. The quality of most studies included was good or very good. Although we identified some high risks of bias, we considered results to be valid as the main data extracted from these studies was from study demographics and not altered by the study.

Another limitation of this review is the heterogeneity due to the different definitions of familial ALS used in the various studies. While there are statistical tests to estimate the extent of statistical heterogeneity, there are no tests to determine the extent of clinical heterogeneity [46]. In our consideration, we determined that meta-analyses would be possible because differences between definitions across studies of familial would be the same in both sexes. Even though participants might be classified differently in another study, this classification applies to both sexes and is therefore expected to have only a marginal impact on sex ratios.

To address inconsistencies in the reporting of familial ALS versus sporadic ALS, a key recommendation would be to implement a consistent clinical definition of familial ALS. For example, the consistent definition can be implemented within the widely accepted El Escorial criteria to diagnose ALS. Frequent updates to these criteria would allow clinicians and researchers to keep up with developing research and the discovery of both novel genes and biomarkers. Another recommendation from this research would be the use of a universal global register for ALS patients. Although some countries have registers, these registers vary in what data they collect. Having universal registers would also allow for consistent reporting that includes different age categories and could allow age and menopausal status to be investigated, as these impact sex ratios in ALS.

Another important consideration for future studies includes the stratification analysis of the sex ratios between the carriers of the well-known familial genes, such as *C9orf76* and *SOD1* and those at-risk genes identified from genome-wide association analysis. A previous meta-analysis [23] reported a higher prevalence of familial ALS among females; however, that study considered only the *C9orf72* gene in its analysis of ALS, which is the most common genetic cause of ALS among European and American populations. The mutation accounts for roughly 30% of the cases (60% in familial ALS and 40% in sporadic ALS) [38]. This suggests familial ALS genes might have a different effect in males and females or are differently expressed due to environmental factors.

This systematic review found that sporadic ALS is more prevalent in males than it is in females, but this is only observed in sporadic ALS. The observed difference in sex ratio between sporadic ALS and familial ALS has significant research and clinical implications. For research, it highlights the need to consider sex-specific risk factors that may uniquely influence sporadic cases, including conducting sex-stratified analyses in clinical trials. Clinically, the disparity in sex ratios raises the possibility that sporadic ALS may be underdiagnosed in females, underscoring the need for increased awareness among healthcare providers.

## Conclusion

ALS is more prevalent in males than it is in females, and our findings suggest sporadic ALS primarily drives this difference. Furthermore, we found that males are more likely to be diagnosed with sporadic ALS compared to females. These findings highlight the importance of considering sex-specific factors in ALS research and clinical practice.

## Supplementary Information

Below is the link to the electronic supplementary material.

Supplementary file1 (DOCX 83 KB)

## Data Availability

Additional supporting information may be found in the Supplementary Materials section.
