# Supplementary material for: The differences in sex ratio between sporadic and familial amyotrophic lateral sclerosis: a systematic review
Source: J Neurol. 2026 Jan 21;273(2):92. doi: 10.1007/s00415-026-13627-1 (PMC12823662; doi:10.1007/s00415-026-13627-1)
Supplement: Supplementary file 1 — Supplementary file1 (DOCX 83 KB) [file 415_2026_13627_MOESM1_ESM.docx]

# upplementary Materials

Table S‑1 MOOSE 2021 Checklist

| **Section and Number** | **Checklist item** | **Page nr** |
| --- | --- | --- |
| **TITLE** | | |
| 1 | Identify the study as a meta-analysis (or systematic review) | 1 |
| **ABSTRACT** | | |
| 2 | Use the journal’s structured format | 1 |
| **INTRODUCTION** | **Present** | |
| 3 | The clinical problem | 2 |
| 4 | The hypothesis | 2 |
| 5 | A statement of objectives that includes the study population, the condition of interest, the exposure or intervention, and the outcome(s) considered | 2 |
| **SOURCES** | **Describe** | |
| 6 | Qualifications of searchers (eg, librarians and investigators) | 3 |
| 7 | Search strategy, including time period included in the synthesis and keywords | 2 |
| 8 | Effort to include all available studies, including contact with authors | 2 |
| 9 | Databases and registries searched | 2 |
| 10 | Search software used, name and version, including special features used (eg, explosion) | 3 |
| 11 | Use of hand searching (eg, reference lists of obtained articles) | 2 |
| 12 | List of citations located and those excluded, including justification | 4, 6-8 |
| 13 | Method of addressing articles published in languages other than English | 3 |
| 14 | Method of handling abstracts and unpublished studies | 3 |
| 15 | Description of any contact with authors | 3 |
| **STUDY SELECTION** | **Describe** | |
| 16 | Types of study designs considered | 3 |
| 17 | Relevance or appropriateness of studies gathered for assessing the hypothesis to be tested | 3 |
| 18 | Rationale for the selection and coding of data (eg, sound clinical principles or convenience) | 4 |
| 19 | Documentation of how data were classified and coded (eg, multiple raters, blinding, and interrater reliability) | 3 |
| 20 | Assessment of confounding (eg, comparability of cases and controls in studies where appropriate) | 5 |
| 21 | Assessment of study quality, including blinding of quality assessors; stratification or regression on possible predictors of study results | 9 |
| 22 | Assessment of heterogeneity | 5 |
| 23 | Statistical methods (eg, complete description of fixed or random effects models, justification of whether the chosen models account for predictors of study results, dose-response models, or cumulative meta-analysis) in sufficient detail to be replicated | 5, 11 |
| **RESULTS** | **Present** | |
| 24 | A graph summarizing individual study estimates and the overall estimate | 5, 11-12 |
| 25 | A table giving descriptive information for each included study | 6-8 |
| 26 | Results of sensitivity testing (eg, subgroup analysis) | 11 |
| 27 | Indication of statistical uncertainty of findings | 11 |
| **DISCUSSION** | **Discuss** | |
| 28 | Strengths and weaknesses | 12-13 |
| 29 | Potential biases in the review process (eg, publication bias) | 13-14 |
| 30 | Justification for exclusion (eg, exclusion of non–English-language citations) | 13 |
| 31 | Assessment of quality of included studies | 13 |
| 32 | Consideration of alternative explanations for observed results | 12 |
| 33 | Generalization of the conclusions (ie, appropriate for the data presented and within the domain of the literature review) | 12 |
| 34 | Guidelines for future research | 13 |
| 35 | Disclosure of funding source | 14 |

*Modified from Stroup DF, Berlin JA, Morton SC, Olkin I, Williamson GD, Rennie D, et al. Meta-analysis of observational studies in epidemiology: a proposal for reporting. Meta-analysis Of Observational Studies in Epidemiology (MOOSE) group. JAMA 2000;283:2008–12. Copyrighted © 2000, American Medical Association. All rights reserved.*

Table S‑2 Search Strategy

| Cochrane Library | |  |
| --- | --- | --- |
|  | |  |
| 1 | Amyotrophic Lateral Sclerosis/ |  |
| 2 | (amyotrophic lateral sclerosis or charcot* disease or gehrig* disease or guam* disease) |  |
| 3 | ((moto* neuron* or motorneuron* or motoneuron*) NEAR/2 disease*) |  |
| 4 | #1 OR #2 OR #3 |  |
| 5 | Sex characteristics/ |  |
| 6 | Female/ |  |
| 7 | Male/ |  |
| 8 | #6 AND #7 |  |
| 9 | (sex* NEAR/2 (differ* or factor* or specific or dimorph* or chromosome* or depend* or link*)) |  |
| 10 | (x chromosome* or y chromosome*) |  |
| 11 | ((male or men) and (female or women)) |  |
| 12 | (gender NEAR/2 difference*) |  |
| 13 | #5 OR #8 OR #9 OR #10 OR #11 OR #12 |  |
| 14 | Heritability |  |
| 15 | exp Genetic Predisposition to Disease/ep, ge, mo [Epidemiology, Genetics, Mortality] |  |
| 16 | Genetics/ or Genetics, Population/ or Genetics, Medical/ |  |
| 17 | “sporadic” |  |
| 18 | “familial” |  |
| 19 | #14 OR #15 OR #16 OR #17 OR #18 |  |
| 20 | #4 AND #13 AND #19 |  |
|  |  |  |
|  |  |  |
| Ovid MEDLINE(R) ALL <1946 to March 26, 2024> | | |
|  | | |
| 1 | | Amyotrophic Lateral Sclerosis/ |
| 2 | | Motor Neuron Disease/ |
| 3 | | (amyotrophic lateral sclerosis or charcot* disease or gehrig* disease or guam* disease).ti,ab,kf. |
| 4 | | ((moto* neuron* or motorneuron* or motoneuron*) adj2 disease*).ti,ab,kf. |
| 5 | | 1 or 2 or 3 or 4 |
| 6 | | Sex characteristics/ |
| 7 | | Sex factors/ |
| 8 | | exp Sex chromosomes/ |
| 9 | | Male/ and Female/ |
| 10 | | (sex* adj3 (differ* or factor* or specific or dimorph* or chromosome* or depend* or link*)).ti,ab,kf. |
| 11 | | (x chromosome* or y chromosome*).ti,ab,kf. |
| 12 | | ((male or men) and (female or women)).ti,ab,kf. |
| 13 | | (gender adj3 difference*).ti,ab,kf. |
| 14 | | 6 or 7 or 8 or 9 or 10 or 11 or 12 or 13 |
| 15 | | Heritability.ti,ab,kf. |
| 16 | | exp Genetic Predisposition to Disease/ep, ge, mo [Epidemiology, Genetics, Mortality] |
| 17 | | Genetics/ or Genetics, Population/ or Genetics, Medical/ |
| 18 | | (Sporadic or Familial).ti,ab,kf. |
| 19 | | 15 or 16 or 17 or 18 |
| 20 | | 5 and 14 and 19 |
|  |  |  |
|  |  |  |
| Embase Classic+Embase <1947 to 2024 March 26> | |  |
|  | |  |
| 1 | Amyotrophic Lateral Sclerosis/ |  |
| 2 | Motor Neuron Disease/ |  |
| 3 | (amyotrophic lateral sclerosis or charcot* disease or gehrig* disease or guam* disease).ti,ab,kf. |  |
| 4 | ((moto* neuron* or motorneuron* or motoneuron*) adj2 disease*).ti,ab,kf. |  |
| 5 | 1 or 2 or 3 or 4 |  |
| 6 | Sex characteristics/ |  |
| 7 | Sex factors/ |  |
| 8 | exp Sex chromosomes/ |  |
| 9 | Male/ and Female/ |  |
| 10 | (sex* adj3 (differ* or factor* or specific or dimorph* or chromosome* or depend* or link*)).ti,ab,kf. |  |
| 11 | (x chromosome* or y chromosome*).ti,ab,kf. |  |
| 12 | ((male or men) and (female or women)).ti,ab,kf. |  |
| 13 | (gender adj3 difference*).ti,ab,kf. |  |
| 14 | 6 or 7 or 8 or 9 or 10 or 11 or 12 or 13 |  |
| 15 | Heritability.ti,ab,kf. |  |
| 16 | exp genetic predisposition/ or exp disease predisposition/ or exp genetic parameters/ |  |
| 17 | Genetics/ or Genetics, Population/ or Genetics, Medical/ |  |
| 18 | (Sporadic or Familial).ti,ab,kf. |  |
| 19 | 15 or 16 or 17 or 18 |  |
| 20 | 5 and 14 and 19 |  |
|  |  |  |
|  |  |  |
| Ovid Emcare <1995 to 2024 Week 12> | |  |
|  | |  |
| 1 | Amyotrophic Lateral Sclerosis/ |  |
| 2 | Motor Neuron Disease/ |  |
| 3 | (amyotrophic lateral sclerosis or charcot* disease or gehrig* disease or guam* disease).ti,ab,kf. |  |
| 4 | ((moto* neuron* or motorneuron* or motoneuron*) adj2 disease*).ti,ab,kf. |  |
| 5 | 1 or 2 or 3 or 4 |  |
| 6 | Sex characteristics/ |  |
| 7 | Sex factors/ |  |
| 8 | exp Sex chromosomes/ |  |
| 9 | Male/ and Female/ |  |
| 10 | (sex* adj3 (differ* or factor* or specific or dimorph* or chromosome* or depend* or link*)).ti,ab,kf. |  |
| 11 | (x chromosome* or y chromosome*).ti,ab,kf. |  |
| 12 | ((male or men) and (female or women)).ti,ab,kf. |  |
| 13 | (gender adj3 difference*).ti,ab,kf. |  |
| 14 | 6 or 7 or 8 or 9 or 10 or 11 or 12 or 13 |  |
| 15 | Heritability.ti,ab,kf. |  |
| 16 | exp genetic predisposition/ or exp disease predisposition/ or exp genetic parameters/ |  |
| 17 | Genetics/ or Genetics, Population/ or Genetics, Medical/ |  |
| 18 | (Sporadic or Familial).ti,ab,kf. |  |
| 19 | 15 or 16 or 17 or 18 |  |
| 20 | 5 and 14 and 19 |  |
|  |  |  |
|  |  |  |
| SCOPUS | |  |
|  | |  |
| ((INDEXTERMS("Amyotrophic Lateral Sclerosis")) OR (INDEXTERMS("Motor Neuron Disease")) OR (TITLE-ABS-KEY("amyotrophic lateral sclerosis" OR "charcot* disease" OR "gehrig* disease")) OR (TITLE-ABS-KEY(("moto* neuron*" OR motorneuron* OR motoneuron*) W/2 disease*))) AND ((INDEXTERMS("Sex Characteristics")) OR (INDEXTERMS("Sex Factors")) OR (INDEXTERMS("Sex Chromosomes")) OR (INDEXTERMS(Male) AND INDEXTERMS(Female)) OR (TITLE-ABS-KEY(sex* W/3 (differ* OR factor* OR specific OR dimorph* OR chromosome* OR depend* OR link*))) OR (TITLE-ABS-KEY("x chromosome*" OR "y chromosome*")) OR (TITLE-ABS-KEY((male OR men) AND (female OR women))) OR (TITLE-ABS-KEY(gender))) AND ((TITLE-ABS-KEY(heritability)) OR (INDEXTERMS(Genetic Predisposition to Disease)) OR (INDEXTERMS(Genetics)) OR (TITLE-ABS-KEY(sporadic OR familial))) | |  |

Figure S 1 Boxplot of male-to-female ratio for familial and sporadic ALS


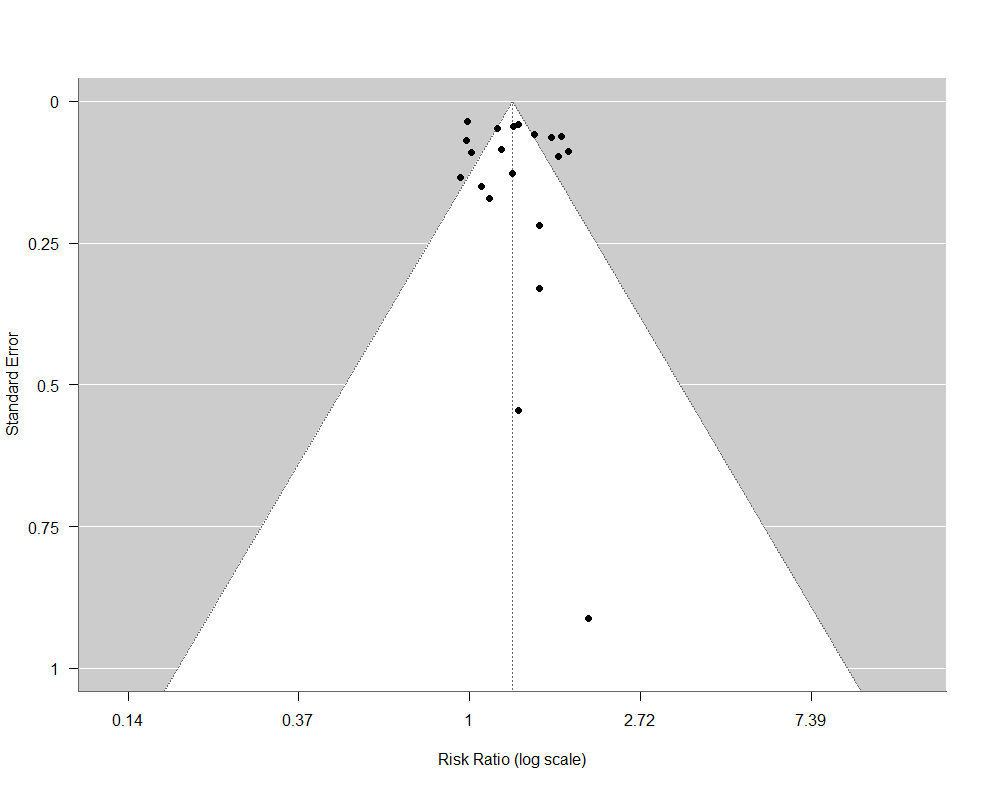


Figure S 2 Funnel Plot using mixed-effects meta-regression model with predictor standard error
